# A Pilot Study on Circadian Activity Rhythm in Pediatric Attention-Deficit Hyperactivity Disorder

**DOI:** 10.3390/clockssleep1030031

**Published:** 2019-08-12

**Authors:** Lorenzo Tonetti, Marina Zoppello, Giorgio Rossi, Umberto Balottin, Marco Fabbri, Marco Filardi, Monica Martoni, Vincenzo Natale

**Affiliations:** 1 Department of Psychology, University of Bologna, Viale Berti Pichat 5, 40127 Bologna, Italy; 2 Child Neuropsychiatry Unit, IRCCS Mondino Foundation, Via Mondino 2, 27100 Pavia, Italy; 3 Direttore Struttura Complessa di Neuropsichiatria dell’Infanzia e dell’Adolescenza, Ospedale Del Ponte, Azienda Socio Sanitaria Territoriale dei Sette Laghi, Via Del Ponte 19, 21100 Varese, Italy; 4 Child Neuropsychiatry Unit, University of Pavia, Via Mondino 2, 27100 Pavia, Italy; 5 Department of Psychology, University of Campania “Luigi Vanvitelli”, Viale Ellittico 31, 81100 Caserta, Italy; 6 Department of Biomedical and Neuromotor Sciences, DIBINEM, University of Bologna, Via Altura 3, 40139 Bologna, Italy; 7 Department of Experimental, Diagnostic and Specialty Medicine, DIMES, University of Bologna, Viale Berti Pichat 5, 40127 Bologna, Italy

**Keywords:** actigraphy, attention-deficit hyperactivity disorder, circadian activity rhythm, functional linear modeling, motor activity, pediatrics

## Abstract

A recent study has applied a novel statistical framework (functional linear modeling: FLM) to the study of circadian activity rhythm (CAR) in adult attention-deficit hyperactivity disorder (ADHD), pointing out the absence of the physiological post-lunch dip. The aim of the present study was to apply FLM to explore the features of CAR in pediatric ADHD. To this end, a secondary analysis of previously collected data was carried out. Twenty-four ADHD children (four females, mean age 8.67 ± 1.74) and 107 controls (C, 60 females, mean age 10.25 ± 0.48) were examined. The actigraph model Actiwatch AW64 was used to objectively monitor sleep/wake behavior and CAR. In the original study each participant wore the actigraph on the non-dominant wrist for one week. FLM was applied to examine the differences between groups in CAR. Compared with C, the CAR of ADHD children was distinguished by a higher motor activity during the whole of the daytime and within a reduced time window during the nighttime.

## 1. Introduction

Attention-deficit hyperactivity disorder (ADHD) is a neurodevelopmental disorder characterized by symptoms of hyperactivity, impulsivity, and inattention. The prevalence of ADHD in childhood and adolescence is high, around 10% [1], with an important persistence into adulthood [2]. Approximately 50% of patients continue to present this disorder.

While excessive motor behavior is an important feature of ADHD, the assessment of this component during the diagnostic process is carried out through standardized scales. One technique that has the potential to provide an objective assessment of motor activity is actigraphy. Actiwatches are wrist-worn devices equipped with an accelerometer that measures the limb activity related to movements occurring during both wake and sleep, i.e., the whole 24 h [3]. Therefore, actigraphy has the unique advantage of providing an objective and naturalistic measure of circadian activity rhythm (CAR). Each epoch of raw motor activity data can also be scored as sleep or wake, according to validated algorithms, providing an indirect assessment of sleep.

The topic of CAR has been previously investigated in both ADHD children and adults (for a review see [4]). Generally speaking, the review cited [4] highlights a circadian phase delay in ADHD patients. More specifically, among the studies on functional assessment of circadian rhythm in ADHD children through actigraphy, reviewed by Coogan and McGowan [4] (see Table 3 from page 137 to page 139), the work by Imeraj and colleagues [5] explored the CAR of combined-type ADHD patients, compared to controls (C), examining the raw motor activity counts hour-by-hour across the 24 h. The authors did not observe differences in motor activity between groups during the nighttime, while higher activity was detected in ADHD patients mainly at noon and during the early afternoon. Dane and colleagues [6] examined motor activity in ADHD children and C for two periods of two hours each, one in the morning and one in the afternoon, during a diagnostic assessment covering a full day. No differences between groups were detected during the morning testing session, while higher motor activity in ADHD children than in C was observed in the afternoon, with no differences emerging between predominantly inattentive and combined subtypes. For the first time, a recent study [7] has applied functional linear modeling (FLM, [8]) to the in-depth analysis of the minute-by-minute CAR in ADHD adults, highlighting the absence of a post-lunch dip as a potential trait marker of ADHD. FLM is a statistical framework specifically developed for the analysis of actigraphic data, and allows an examination of functional (not row) form of minute-by-minute CAR; it adopts both a categorical and continuous approach, through a non-parametric permutation F-test. Since FLM examines data in their natural form (i.e., actigraphic time series), more information can be extracted than that derived through the conversion of data into average activity measures, which are then analyzed by classical statistical approaches. In addition to CAR of ADHD adults [7], FLM has allowed researchers to successfully describe the CAR of pediatric narcolepsy type 1 [9] and adult affective disorder [10] patients. 

To the best of our knowledge, no studies have yet applied FLM to describe the minute-by-minute CAR of ADHD children. For this reason, we chose to fill this gap of knowledge by carrying out a secondary analysis of previous data, collected separately in ADHD and C children, with the aim of assessing whether the potential ADHD trait marker detected in adults may already be present in children.

## 2. Results

With reference to actigraphic sleep parameters, we found two significant differences between the groups (Table 1), with longer wake after sleep onset (WASO) and lower sleep efficiency (SE) in ADHD children compared to C. As regards gender and interaction between factors, no significant effects were detected (Table 1).

**Figure 1:**
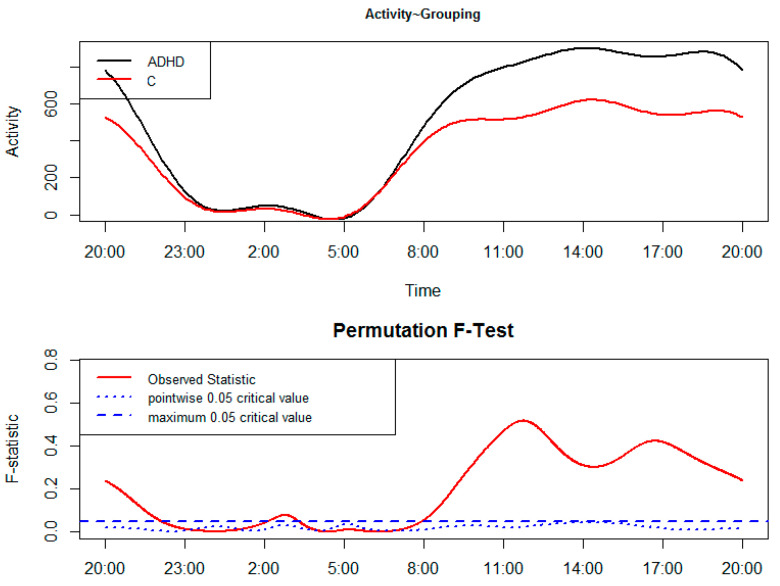
Functional linear modeling applied to the group comparison between attention-deficit hyperactivity disorder (ADHD) children and controls (C).

**Figure 2:**
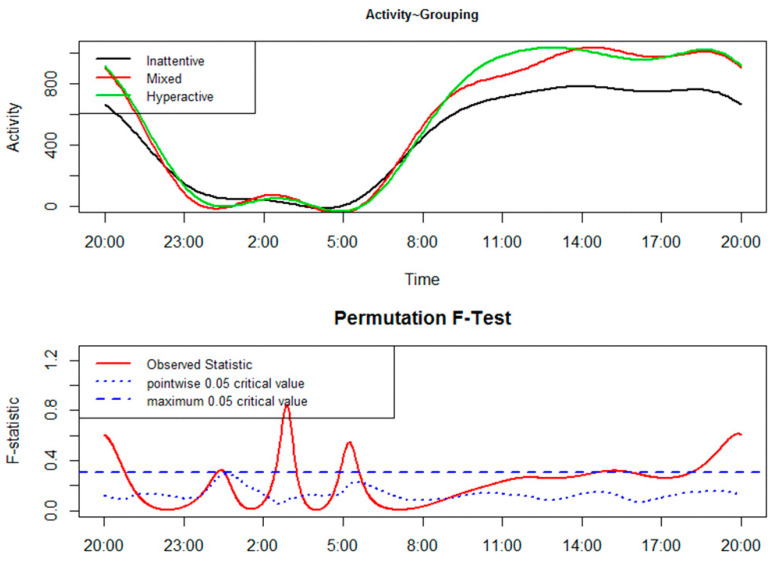
Functional linear modeling applied to the comparison between children belonging to predominantly inattentive-, predominantly hyperactive/impulsive-, and combined-subtype.

The results of the FLM are reported in Figure 1 and Figure 2. In the upper panel the functional forms of CAR of groups are reported, while the lower panel shows the results of the permutation F-test. From a statistical point of view, CARs of groups significantly differ when the red solid line (i.e., the observed statistic) is above the blue dashed line (i.e., the global test of significance with alpha set to 0.05).

Figure 1 shows the comparison between CAR of ADHD children and C, with patients moving significantly more than C from around 8:00 to 22:00 and again from about 02:00 to 03:00. 

Figure 2 shows the group comparison between children belonging to the three subtypes of ADHD. Children with predominantly hyperactive/impulsive- and combined-subtypes moved significantly more than those with a predominantly inattentive-subtype from around 18:00 to 21:00 and at about 03:00. Around 05:00, children with a predominantly inattentive-subtype moved significantly more than those of the other two subtypes.

## 3. Discussion

The aim of this secondary analysis of previously collected data was to explore, for the first time, the features of CAR in pediatric ADHD through FLM. In this case CAR refers to the 24-h time course of motor activity.

Data on actigraphic sleep parameters, reported in Table 1, globally show that ADHD children present an impaired actigraphic sleep quality, in comparison to C, in terms of a reduced SE and an extended WASO. These findings are, overall, in line with recent evidence on this topic [11,12].

The results of the FLM applied to a group comparison between ADHD children and C (Figure 1) point to a motor hyperactivity in patients, which covers almost the whole of the daytime (i.e., the actigraphic time spent out of bed) and is also evident within a reduced time window during the nighttime. The peculiar hyperactivity observed in ADHD children during a specific nighttime period (from 02:00 to 03:00) could be potentially viewed as a by-product of low sleep quality. Taking into account the two-process model of sleep regulation [13], it is as if homeostatic sleep pressure is not enough to preserve sleep continuity. Although different in methodological and statistical approach, the results of our study are overall in line with those reported in ADHD children by Imeraj and colleagues [5]. In comparison with recent FLM data observed in adults with ADHD (i.e., hyperactivity in patients from 4:00 to 7:00 and from 12:00 to 18:00; [7]), the children’s motor hyperactivity is more extended during the daytime and less so during the nighttime, leading to the suggestion that age could actually modulate the features of CAR. Indeed, in children motor hyperactivity was observed in 15 out of the 24 h, while in adults it was apparent for an overall amount of 9 h [7], probably pointing toward a more severe hyperactivity during childhood that may be smoothed with aging due, for example, to pharmacological or behavioral treatments. From our point of view, it is also interesting to highlight that the post-lunch dip seems less marked in patients than C, in line with previous evidence in adults [7], pointing out that the role of a smoothed/absent post-lunch dip as a marker of ADHD definitely deserves further in-depth analysis. This less marked post-lunch dip in ADHD children can be observed in Figure 1 from around 14:00 to 16:00, with a slighter decrease in motor activity compared to C.

Within the ADHD group, the comparison between children belonging to the three ADHD subtypes (Figure 2) is potentially interesting because it has shown that actigraphy, through a detailed analysis of CAR with FLM, could also be a useful technique to discriminate between the subtypes. However, we also have to acknowledge that the results obtained are weak and should be treated with caution due to the small size of the three ADHD subtype groups. 

Some limitations of the present study should be acknowledged. In particular, the size of the sample of ADHD children is slightly lower compared to previous studies on a similar topic (e.g., 30 patients in [5]). Moreover, ADHD children and C were different for demographic features of gender and age. Finally, psychiatric disorders were not assessed in the C group.

## 4. Materials and Methods

### 4.1. Participants

In the present work, we examined 24 ADHD children (four females; age range: 5–12 years; median age: 9 years; and age mode: bimodal, 8 and 9 years) who were being treated at the Child Neuropsychiatry Unit of the Fondazione Mondino (Istituto Neurologico Nazionale a Carattere Scientifico | IRCCS (Pavia, Italy)), and who participated in a study on actigraphic sleep quality and raw hourly CAR between 2004 and 2006, the results of which have previously been reported as abstracts [14,15]. Patients were recruited by staff members of the Child Neuropsychiatry Unit who approached the parents to propose the children’s participation in the study. The exclusion criterion of total intelligence quotient (IQ) score lower than 90 was applied by administering the Wechsler Intelligence Scale for Children-Revised (WISC-R) [16]. Prior to the original study, an experienced child neuropsychiatrist had put forward the diagnosis of ADHD according to the criteria of Diagnostic and Statistical Manual of Mental Disorders (DSM-IV-TR, [17]). Twelve patients presented the predominantly inattentive subtype, 10 had the combined subtype, and two were of the predominantly hyperactive/impulsive subtype. Fourteen patients presented a comorbidity; of these, 10 presented a comorbidity with specific learning disorders, two with oppositional defiant disorder, and two with both specific learning disorders and oppositional defiant disorder. At the time of actigraphic recording, no patients were taking medications [18]. 

In this secondary analysis of previous data, we also examined 107 C (60 females; age range: 9–11.80 years; median age: 10 years; and age mode: 10 years) who had been enrolled in a study on the relationship between body mass index and sleep/wake cycle, assessed through actigraphy [19], conducted between 2013 and 2014. C took part in the original study [19] only if they did not meet the following exclusion criteria: sleep disorders, severe or acute disease, disabilities interfering with or restricting motor behavior, and consumption of psychopharmaceutical medications. These exclusion criteria were ruled out through a semi-structured interview carried out by a trained researcher. C were attending the fourth and fifth year of primary schools located in the city of Bologna (Italy) and in the districts of its province as well as in the town of Castelfranco Emilia (province of Modena, Italy) and its hamlets. 

The distribution of gender across the groups of ADHD and C was significantly different (χ^2^_1_ = 12.18; *p* < 0.001), with a higher prevalence of males among the ADHD group. The groups also significantly differed by age, which was lower in ADHD (8.67 ± 1.74) than C (10.25 ± 0.48) (t_129_ = −8.21; *p* < 0.001).

### 4.2. Actigraphy

The Actiwatch AW64 (Cambridge Neurotechnology Ltd, UK) was used to objectively assess sleep and CAR. Using the software Actiwatch Activity and Sleep Analysis 5 (version 5.32), each epoch was scored as sleep or wake according to the algorithm proposed by Oakley [20], setting the wake sensitivity to low [21,22].

The same software was used to extract minute-by-minute raw motor activity counts across the 24 h, in order to draw the raw CAR.

### 4.3. Actigraphic Sleep Parameters

The following actigraphic parameters were calculated: bedtime (BT), the clock time at which participants went to bed and tried to fall asleep; get-up time (GUT), the clock time at which children got out of bed after nocturnal sleep; time in bed (TIB), the interval in minutes between BT and GUT; midpoint of sleep (MS), the clock time that splits TIB in half; sleep onset latency (SOL), the interval in minutes between BT and sleep start (SS); total sleep time (TST), the sum, in minutes, of all sleep epochs between SS and GUT; wake after sleep onset (WASO), the sum, in minutes, of all wake epochs between SS and GUT; sleep efficiency (SE), the ratio between TST and TIB, multiplied by 100; mean activity score (MAS), the average value of the activity counts per epoch over the assumed sleep period; and wake bouts (WB), the number of episodes of sleep interruptions with wake periods of at least 1 min.

### 4.4. Procedure

In the original study, ADHD children and C had been requested to wear the actigraph around the non-dominant wrist for one week [23]. While all ADHD patients complied with this request, 57 C out of 164 were excluded because of technical problems occurring during the actigraphic recording, because they chose to retire from the study, or because fewer than five school days were recorded. Participants had to press the event-marker button on the actigraph at BT and GUT, allowing the scorer to set the TIB for actigraphic analysis on sleep. In cases where they failed to press the event-marker button, the scorer referred to the replies to questions on BT and GUT in a simplified sleep diary, which was filled in daily within 30 min from GUT with the help of parents. Parents of both ADHD children and C provided written informed consent prior to the beginning of actigraphic recording of their child.

In the present study, the actigraphic sleep parameters of school nights only were computed. Similarly, in order to depict the raw circadian motor activity profile, only school days were examined. To this aim, we visually inspected the actigraphy files to identify and exclude from the analysis the time periods in which children removed the actigraph, as reported in the simplified sleep diary.

The study was approved by the Bioethics Committee of the University of Bologna (Bologna, Italy; report of 11.9.2013).

### 4.5. Statistical Analyses

With reference to each actigraphic sleep parameter, treated as a dependent variable, we performed a univariate analysis of covariance (ANCOVA) with gender (two levels: males and females) and group (two levels: ADHD children and C) as fixed factors and age as a covariate. Since we performed multiple comparisons, we corrected the significance level through Bonferroni, considering p values less than 0.005 as significant.

With the aim of assessing if and when the CAR of groups significantly differed across the 24 h, we applied FLM through the routine “Actigraphy” implemented within the statistical software R [24]. First, FLM transforms the raw circadian motor activity profile into a function by applying the Fourier expansion model fitted at a periodicity of 24 h. Second, FLM applies the non-parametric permutation F-test aiming to assess, minute-by-minute over the 24 h, if and when the CAR of groups significantly differed. We applied FLM to two between-groups comparisons: (1) ADHD children versus C and (2) within the ADHD group, a comparison between predominantly inattentive-, predominantly hyperactive/impulsive-, and combined-subtype. 

## 5. Conclusions

This work was a pilot study on CAR in pediatric ADHD. In this instance CAR pertained to the 24-h motor activity time course. The main findings were the motor hyperactivity observed just in one specific time window during the nighttime (i.e., from 02:00 to 03:00) and a less marked post-lunch dip (a potential marker of the disorder) in ADHD children than C, that seemed to point out a lower homeostatic sleep pressure in pediatric ADHD. Furthermore, such study showed preliminary but promising findings about the usefulness of actigraphy in the discrimination between ADHD subtypes. 

It would be extremely interesting if future studies could thoroughly examine the CAR of children at the time of the first referral to child neuropsychiatric units for behavioral or attentional problems, before a formal diagnosis of ADHD is made and prior to any type of treatment. This kind of study would allow researchers to explore the possible role of a smoothed/absent post-lunch dip, as a marker of this disorder, more thoroughly, and in a more concrete manner.

## Figures and Tables

**Table 1:** Estimated marginal means and standard errors of actigraphic sleep parameters according to gender (M and F), group (ADHD children and C), and their interaction. Statistics are also reported, with significant differences marked in bold.

Actigraphic Sleep Parameter		ADHD Children	C	Mean by Gender	Statistics ^a^
BT	M	22:13 ± 0:11	22:17 ± 0:06	22:15 ± 0:06	Gender: F_1,126_ = 0.46; *p* = 0.50
	F	21:53 ± 0:22	22:21 ± 0:05	22:07 ± 0:11	Group: F_1,126_ = 1.29; *p* = 0.26
Mean by group		22:03 ± 0:13	22:19 ± 0:04		Interaction: F_1,126_ = 1.08; *p* = 0.30
GUT	M	07:34 ± 0:08	07:30 ± 0:05	07:32 ± 0:05	Gender: F_1,126_ = 0.02; *p* = 0.88
	F	07:33 ± 0:17	07:34 ± 0:04	07:34 ± 0:08	Group: F_1,126_ = 0.01; *p* = 0.92
Mean by group		07:34 ± 0:10	07:32 ± 0:04		Interaction: F_1,126_ = 0.08; *p* = 0.77
TIB	M	560.59 ± 9.23	553.70 ± 5.34	557.14 ± 5.01	Gender: F_1,126_ = 0.70; *p* = 0.40
	F	579.99 ± 18.20	551.49 ± 4.63	565.74 ± 9.30	Group: F_1,126_ = 2.27; *p* = 0.13
Mean by group		570.29 ± 10.71	552.60 ± 3.61		Interaction: F_1,126_ = 1.11; *p* = 0.30
MS	M	02:54 ± 0:08	02:53 ± 0:05	02:53 ± 0:05	Gender: F_1,126_ = 0.13; *p* = 0.72
	F	02:43 ± 0:17	02:58 ± 0:04	02:50 ± 0:08	Group: F_1,126_ = 0.45; *p* = 0.50
Mean by group		02:48 ± 0:10	02:56 ± 0:04		Interaction: F_1,126_ = 0.64; *p* = 0.42
TST	M	475.30 ± 8.56	485.73 ± 4.95	480.52 ± 4.64	Gender: F_1,126_ = 1.47; *p* = 0.23
	F	487.90 ± 16.87	496.23 ± 4.29	492.07 ± 8.62	Group: F_1,126_ = 0.74; *p* = 0.39
Mean by group		481.60 ± 9.93	490.98 ± 3.35		Interaction: F_1,126_ = 0.01; *p* = 0.91
SE	M	84.88 ± 0.96	87.67 ± 0.56	86.28 ± 0.52	Gender: F_1,126_ = 0.18; *p* = 0.68
	F	83.68 ± 1.90	89.77 ± 0.48	86.73 ± 0.97	Group: **F_1,126_ = 13.19; *p* < 0.001**
Mean by group		84.28 ± 1.12	88.72 ± 0.38		Interaction: F_1,126_ = 2.37; *p* = 0.13
SOL	M	22.21 ± 2.79	17.92 ± 1.61	20.06 ± 1.51	Gender: F_1,126_ = 0.75; *p* = 0.39
	F	22.58 ± 5.50	12.16 ± 1.40	17.37 ± 2.81	Group: F_1,126_ = 4.30; *p* = 0.04
Mean by group		22.39 ± 3.24	15.04 ± 1.09		Interaction: F_1,126_ = 0.98; *p* = 0.33
WB	M	27.35 ± 1.65	24.92 ± 0.95	26.13 ± 0.89	Gender: F_1,126_ = 0.89; *p* = 0.35
	F	31.89 ± 3.25	23.84 ± 0.83	27.86 ± 1.66	Group: F_1,126_ = 6.25; *p* = 0.01
Mean by group		29.62 ± 1.91	24.38 ± 0.64		Interaction: F_1,126_ = 2.35; *p* = 0.13
MAS	M	24.44 ± 2.66	16.57 ± 1.54	20.51 ± 1.44	Gender: F_1,126_ = 0.24; *p* = 0.62
	F	24.11 ± 5.25	13.99 ± 1.33	19.05 ± 2.68	Group: F_1,126_ = 7.06; *p* = 0.009
Mean by group		24.28 ± 3.09	15.28 ± 1.04		Interaction: F_1,126_ = 0.14; *p* = 0.71
WASO	M	52.51 ± 4.11	43.30 ± 2.37	47.91 ± 2.23	Gender: F_1,126_ = 0.26; *p* = 0.61
	F	61.79 ± 8.09	38.67 ± 2.06	50.23 ± 4.13	Group: **F_1,126_ = 9.58; *p* < 0.005**
Mean by group		57.15 ± 4.76	40.99 ± 1.61		Interaction: F_1,126_ = 2.32; *p* = 0.13

ADHD = attention-deficit hyperactivity disorder, C = controls, M = males, F = females, BT = bedtime (h:min), GUT = get-up time (h:min), TIB = time in bed (min), MS = midpoint of sleep (h:min), TST = total sleep time (min), SE = sleep efficiency (%), SOL = sleep onset latency (min), WB = wake bouts (number), MAS = mean activity score (activity counts), and WASO = wake after sleep onset (min). ^a^ Significance level was set to *p* < 0.005 after Bonferroni correction.

## Abbreviations

FLM	Functional Linear Modeling
CAR	Circadian activity rhythm
ADHD	Attention-deficit hyperactivity disorder
C	Controls
WASO	Wake after sleep onset
SE	Sleep efficiency
M	Males
F	Females
BT	Bedtime
GUT	Get-up time
TIB	Time in bed
MS	Midpoint of sleep
TST	Total sleep time
SOL	Sleep onset latency
WB	Wake bouts
MAS	Mean activity score
IQ	Intelligence quotient
WISC-R	Wechsler Intelligence Scale for Children-revised
DSM-IV-TR	Diagnostic and Statistical Manual of Mental Disorders
SS	Sleep start
ANCOVA	Analysis of covariance
